# Insights into small-molecule compound CY-158-11 antibacterial activity against *Staphylococcus aureus*

**DOI:** 10.1128/msphere.00643-24

**Published:** 2024-09-23

**Authors:** Li Shen, Junhong Shi, Weihua Han, Jingyi Yu, Xinru Yuan, Haojin Gao, Yu Huang, Jianbo Lv, Cailing Wan, Peiyao Zhou, Yanghua Xiao, Jiao Zhang, Bingjie Wang, Rongrong Hu, Fangyou Yu

**Affiliations:** 1 Department of Clinical Laboratory, Shanghai Pulmonary Hospital, School of Medicine, Tongji University, Shanghai, China; 2 Shanghai Institute of Immunity and Infection Chinese Academy of Science, Shanghai, China; University of Nebraska Medical Center College of Medicine, Omaha, Nebraska, USA

**Keywords:** *Staphylococcus aureus*, CY-158-11, antibacterial activity, cell membrane, skin abscess

## Abstract

The widespread prevalence and dissemination of antibiotic-resistant bacteria, coupled with the diminishing supply of new antibiotics, emphasize the pressing necessity for the exploration of innovative antibacterial agents. Previously, we detailed the impact of the small-molecule compound CY-158-11 on *S. aureus* biofilm. By hindering adhesion and PIA-mediated biofilm formation, subinhibitory concentrations of CY-158-11 exhibit antibiofilm activity toward *S. aureus*. Here, we sought to elucidate the antibacterial activity and mode of action of this compound. Upon CY-158-11 treatment in culture, the inhibition of bacterial growth, coupled with MBC to MIC of >4, indicated that CY-158-11 exerted a bacteriostatic effect. Particularly, CY-158-11 showed strong antibacterial activity against a wide variety of *S. aureus*, including multidrug-resistant bacteria. We found that CY-158-11 promoted the permeability of cell membrane and propidium iodide absorption as well as caused the dissipation of membrane potential. The effect of CY-158-11 on the mammalian cytoplasmic membrane was measured using hemolytic and cytotoxicity assays, and the skin irritation and systemic toxicity of the drug were measured by injecting the compound into the skin and tail vein of mice. Moreover, CY-158-11 exhibited considerable efficacy in a subcutaneous abscess mouse model of *S. aureus* infection. In conclusion, CY-158-11 possesses antibacterial properties, including inhibition of bacterial growth, damage to cell membranes, and treatment of skin abscesses, which can be a promising therapeutic option for combating *S. aureus*.

**IMPORTANCE:**

The combination of the rising incidence of antibiotic resistance and the shrinking antibiotic pipeline has raised concern about the postantibiotic era. New antibacterial agents and targets are required to combat *S. aureus*-associated infections. In this study, we identified a maleimide-diselenide hybrid compound CY-158-11 exhibiting antibacterial activity against *S. aureus in vitro* and *in vivo* at relatively low concentrations. Furthermore, the investigation of its mode of action revealed that CY-158-11 can selectively perturb the cytoplasmic membrane of bacteria without harming mammalian cells or mouse organs. Thus, CY-158-11 is a compelling novel drug for development as a new therapy for *S. aureus* infections.

## INTRODUCTION

*Staphylococcus aureus* is a prominent gram-positive opportunistic human pathogen that encompasses a broad spectrum of clinical infections. Approximately 30% of the human population is colonized with *S. aureus*, which serves as a primary etiological agent for various conditions, ranging from skin and soft tissue infections to sepsis and meningitis (1–3). The widespread and indiscriminate use of antibiotics for bacterial infections has led to the development and spread of antibiotic resistance, which poses more challenges in treating *S. aureus* infections and renders longer hospitalization and higher mortality rates for patients (4). Most of the currently known antibiotics confer antibacterial activities by impeding essential processes in bacterial cells, for example, translation, transcription, replication, or cell wall synthesis (5). However, bacteria have indeed developed various resistance mechanisms to evade attacks that target those pathways (6), resulting in high clinical failure rates of antibiotic therapy. Because of the probability for exposed bacteria to generate resistance phenotypes, coupled with the situation that the WHO has prioritized *S. aureus* as a great threat to public health, there is an urgent need to develop novel antibacterial agents that attack targets with different modes of action.

The bacterial cytoplasmic membrane is a crucial component that not only maintains the integrity of the cell but also plays a central role in bacterial physiology, metabolism, and adaptation to various environmental conditions (7). Accordingly, any disruption or damage to this membrane can have a profound impact on bacterial viability. Antibiotics that target the cytoplasmic membrane can disrupt its integrity, leading to the leakage of essential intracellular components and the loss of membrane potential, ultimately leading to bacterial cell death (8). The conservative nature of the cytoplasmic membrane makes it less prone to mutation and resistance development. In contrast, other bacterial targets, such as enzymes or protein synthesis machinery, can undergo mutations and result in antibiotic resistance. Due to its conservative structure and essential functions in bacterial physiology, the cytoplasmic membrane represents a promising target for the development of new antibiotics (9).

CY-158-11 is indeed a small-molecule compound composed of N-maleimide and diphenyl diselenide. Maleimide is a versatile compound widely used in biomedicine, organic synthesis, and materials science (10, 11), while diphenyl diselenide is an organoselenium compound with various biological activities (12). The combination of these two components in CY-158-11 likely imparts unique chemical and biological properties to the compound, making it of interest for research and potential applications in areas such as drug discovery. Previously, we reported that the small-molecule compound CY-158-11 exerted its inhibitory effects against the biofilm formation in *S. aureus* (13). Herein, we further investigated the potent antibacterial activity of CY-158-11 against *S. aureus*, considering its lower MIC. Our study focused on the mode of action and efficacy of CY-158-11. We did not observe any innate or acquired resistance of *S. aureus* to CY-158-11 through a selection of drug-resistant *S. aureus* isolates. Further analysis of CY-158-11’s impact on the bacterial cells indicated that this compound likely targets the bacterial membrane. The therapeutic efficacy of the identified compound was validated using murine models of skin wound infection induced by *S. aureus*. Thus, the unique mechanism of action of CY-158-11 presents an opportunity to develop synthetic compounds as an antibacterial agent that can reduce the likelihood of bacterial resistance.

## MATERIALS AND METHODS

### Bacterial strains

The type strains, including *S. aureus* ATCC 25923, ATCC 29213, Newman, NCTC8325, Mu50, MW2, N315, and USA300, *S. epidermidis* RP62A and ATCC 12228, *K. pneumoniae* NTUH-K2044, *P. aeruginosa* PAO1, and *E. coli* ATCC 25922, were stored in our laboratory. Clinical strains of *S. aureus* were isolated from the first affiliated hospital of Wenzhou Medical University (JP21 and JP30) and Renji Hospital, Shanghai Jiao Tong University School of Medicine (MR255). SA113 was kindly provided by the Department of Infectious Diseases and the Key Laboratory of Endogenous Infection, Shenzhen Nanshan People’s Hospital. Clinical strain *S. epidermidis* SE1457 was preserved in the laboratory. *S. aureus* and *S. epidermidis* strains were grown in tryptic soy broth (TSB). Gram-negative species were grown in Luria Bertani broth. All bacteria were grown at 37°C, 220 rpm. Bacterial strains JP21 and JP30 used in this study were described at Table 1.

**TABLE 1:** Bacterial strains used in this study^*a*^

Strain	Source	MLST	*spa* type	Antibiotic resistance/susceptibility profile
JP21	Tissue	ST7	t091	OX(S); P(S); E(R); DA(R); TE(R); CIP(S); LEV(S); MXF(S); CN(S); VA(S); LZD(S)
JP30	Sputum	ST5	t2460	OX(R); P(R); E(R); DA(R); TE(R); CIP(R); LEV(R); MXF(R); CN(R); VA(S); LZD(S)

^*a*^ OX, oxacillin; P, penicillin; E, erythromycin; DA, clindamycin; TE, tetracycline; CIP, ciprofloxacin; LEV, levofloxacin; MXF, moxifloxacin; CN, gentamicin; VA, vancomycin; LZD, linezolid.

### Cell lines

The human lung cancer cell line A549 was purchased from the American Type Culture Collection (Rockville, Maryland, USA). The human leukemia monocytic cell line THP-1 was obtained from the Cell Bank of the Type Culture Collection of the Chinese Academy of Sciences. A549 cells were cultured in DMEM medium supplemented with 10% FBS and 1% penicillin/streptomycin solution. THP-1 cells were cultured in RPMI 1640 medium supplemented with 10% FBS and 1% penicillin/streptomycin solution. All cells were maintained at 37°C in a 5% CO_2_ incubator.

### MIC and MBC assays

The bacteria were grown to the mid-logarithmic phase, and 1.5 × 10^6^ CFU/mL cells were diluted into cation-regulated Mueller-Hinton broth (CAMHB). Twofold serial dilutions of CY-158-11 (2–256 µg/mL) were mixed with the same volume (100 µL) of the bacterial suspensions in 96-well microtiter plates. After 16–18 h of incubation at 37°C, the MICs were defined as the amount of antibacterial that inhibits visible growth of the microorganism. The mixture was removed 100 µL from each well that did not exhibit visual bacteria growth and then subcultured on agar plates followed by incubation for 24 h at 37°C. The MBCs were the lowest concentration of an antibacterial agent that either totally prevents growth or results in a ≥99.9% decrease in the initial inoculum on subculture.

### Growth curves

An overnight culture of *S. aureus* was adjusted to the equivalent of 0.5 McFarland standard and diluted 1:100 in the tube containing TSB. Next, the bacteria cell suspension was treated with different concentrations of CY-158-11 at 1/2× MIC, 1× MIC, 2× MIC, and 4× MIC, respectively. DMSO at 4× MIC was used as a control. The value of OD_600_ every 1 h for 24 h was measured by using an automatic microbial growth curve analyzer (OY Growth Curves, Finland). The growth curve was generated according to the measured values.

### Time-kill curves

Tubes containing TSB were inoculated with a suspension of *S. aureus* to a final count of approximately 1 × 10^6^ CFU/mL. Then the bacterial suspension (5 mL) was challenged with CY-158-11 at 1/2× MIC, 1× MIC, 2× MIC, and 4× MIC. Subsequently, the mixtures were cultured at 37°C and 220 rpm. At 0, 2, 4, 8, 12, and 24 h, 100 µL aliquots were removed to be serially diluted 10-fold in phosphate-buffered saline (PBS) and plated on TSA plates. After overnight incubation at 37°C, the CFU was evaluated by plate counts.

### Bacterial resistance studies

For resistance development by sequential passaging, *S. aureus* at the exponential phase was passaged to new TSB containing CY-158-11, vancomycin, or linezolid at concentrations of 0.5× MIC. Following a 24 h of incubation at 37°C, the bacterial suspensions were then re-passaged for the next MIC assay. The alterations in MIC values were monitored over a period of 20 days. The results were presented as a fold increase in MIC concerning the initial MIC value.

### Membrane integrity analysis

Membrane damage was measured by detecting intracellular leakage. Mid-log phase *S. aureus* (approximately 1 × 10^8^ CFU/mL) was exposed to 1× MIC, 2× MIC, and 4× MIC of CY-158-11. The bacteria without treatment were used as negative controls. After 4 h of incubation, the bacterial suspension was diluted 1:10 with PBS and stained with 10 µg/mL propidium iodide (PI, Thermo Fisher Scientific, Waltham, MA, USA), a membrane-impermeable dye, for 30 min in the dark. The samples were analyzed using a FACS Calibur Flow Cytometer (BD, USA) equipped with a 488 nm laser. Gates were drawn based on the untreated controls (PI-negative).

### Membrane potential analysis

Membrane depolarization was measured using the membrane potential-sensitive dye 3,3′-diethyloxacarbocyanine iodide [DiOC_2_(3), Sigma-Aldrich]. Mid-log phase *S. aureus* was diluted to 1 × 10^8^ CFU/mL and then incubated with CY-158-11 (1×, 2×, and 4× MIC) for 4 h at 37°C. The cells treated with 5 µM of the proton ionophore carbonyl cyanide 3-chlorophenylhydrazone (CCCP, MCE) were used as depolarized control. And non-treated cells were used as a polarized control. DiOC_2_(3) was added to the bacterial suspension (approximately 1 × 10^7^ CFU/mL) at a final concentration of 30 µM. The fluorescence signals of cells were determined by FACS Calibur Flow Cytometer (BD, USA). Gates were drawn based on the CCCP-treated controls. The membrane potential was calculated from the ratio of red and green fluorescence.

### Transmission electron microscopy

*S. aureus* was grown to exponential phase at 37°C, 200 rpm. The bacterial cells were treated with CY-158-11 at 16 µg/mL (4× MIC) for 4 h. After cell collection by centrifugation, the cells were fixed in 2.5% glutaraldehyde overnight at 4°C. The fixed cells were washed in 0.1 M PB buffer and postfixed with 1% osmium tetroxide. After rinsing with ddH_2_O, the cells were incubated in 1% uranyl acetate. Then the cells were dehydrated with gradient concentrations of ethanol (50%, 70%, 90%, and 100%) for 10 min each and placed in ethanol and acetone solution (1:1) for 5 min each. Subsequently, the cells were infiltrated in a mixture of acetone and resin. After embedding in the resin, the cells were polymerized and dried. Ultrathin sections (approximately 70 nm) were cut on a Reichert Ultracut-S microtome, picked up onto copper grids, and stained with lead citrate. Micrographs of the cells were taken using a transmission electron microscope (HITACHI HT-7700, Japan).

### Hemolysis assay

Fresh human red blood cells (hRBCs, *n* = 3 per condition) were used to evaluate the hemolytic activity of CY-158-11. hRBCs were rinsed with PBS three times and suspended to a final concentration of 8% (vol/vol). An equal volume of hRBC suspensions was then exposed to DMSO (mock), 1% Triton X-100, or CY-158-11 at increasing doses (ranging from 8 to 256 µg/mL). After incubation at 37°C for 1 h, the mixtures were centrifuged at 4,000 rpm for 5 min, and the absorbance of the supernatants was measured at 600 nm. Hemolysis (%) = [(absorbance of the treated sample − absorbance of the negative control)] ÷ [(absorbance of the positive control − absorbance of the negative control)] × 100%. The HC50 values were calculated using the concentration of the tested compound required to lyse 50% of red blood cells. The selectivity index (SI) was calculated from HC50 (for hRBCs) divided by MIC of *S. aureus* strains (SI = HC50/MIC).

### Cytotoxicity assay

A549 cells and THP-1 cells were used to assess the safety of CY-158-11 *in vitro*. Cells (1 × 10^4^ cells/well) were treated with concentrations of CY-158-11 (8–256 µg/mL). The wells containing the solvent (DMSO) were used as a control group. After incubation at 37°C for 24 h, a CCK-8 kit was used to determine the cell activity according to the manufacturer’s instructions. The absorbance of each well was recorded by the microplate reader at 450 nm. Cell viability (%) = [(absorbance of the drug treatment group − absorbance of blank)] ÷ [(absorbance of the non-drug treatment group − absorbance of blank)] × 100%. In this study, the IC50 represents the concentration of the compound that is required for 50% inhibition of a cell. The SI was calculated from IC50 (for THP-1 and A549 cells) divided by MIC of *S. aureus* strains (SI = IC50/MIC).

### Animal studies

BALB/C female mice, aged 6 weeks and weighing 18–20 g, were used. To examine the safety of the compound *in vivo*, we injected 100 µL 16 µg/mL CY-158-11 into the skin or tail vein of mice every 24 h for 3 days. Normal saline containing an equivalent volume of DMSO was used as a negative control. On the fourth day, the skin or internal organs of one mouse in each group were taken for a pathological section.

To explore the *in vivo* antibacterial effect of CY-158-11, we built the mouse skin abscess model. The mice were randomly divided into five groups (*n* = 6 per group). *S. aureus* (0.5 McFarland standard) in the mid-log phase were injected into the mice subcutaneously. At 1 h post-infection, 0.08 mg/kg (4× MIC) CY-158-11 was applied directly to the infected area. To compare the results with CY-158-11, we utilized 0.01 mg/kg (4× MIC) vancomycin with the animal models of infection, and the vehicle (0.1% DMSO) was applied to the skin as a control. All drugs and normal saline were administered to the wounds every 24 h for 3 days. The areas of skin abscesses were recorded daily using a caliper. On the fourth day, the skin was excised and homogenized in 1 mL of normal saline. The number of viable cells was counted.

### Statistical analysis

All experimental data represent mean ± standard deviation (SD). All figures were obtained from several independent experiments, which showed similar results. All data were analyzed using GraphPad Prism 9.0 software. To examine the significant differences between groups, the data were compared using a Student *t*-test. *, *P* < 0.05; **, *P* < 0.01; ***, *P* < 0.001; ****, *P* < 0.0001.

## RESULTS

### Characterization of small-molecule compound CY-158-11

**Fig 1:**
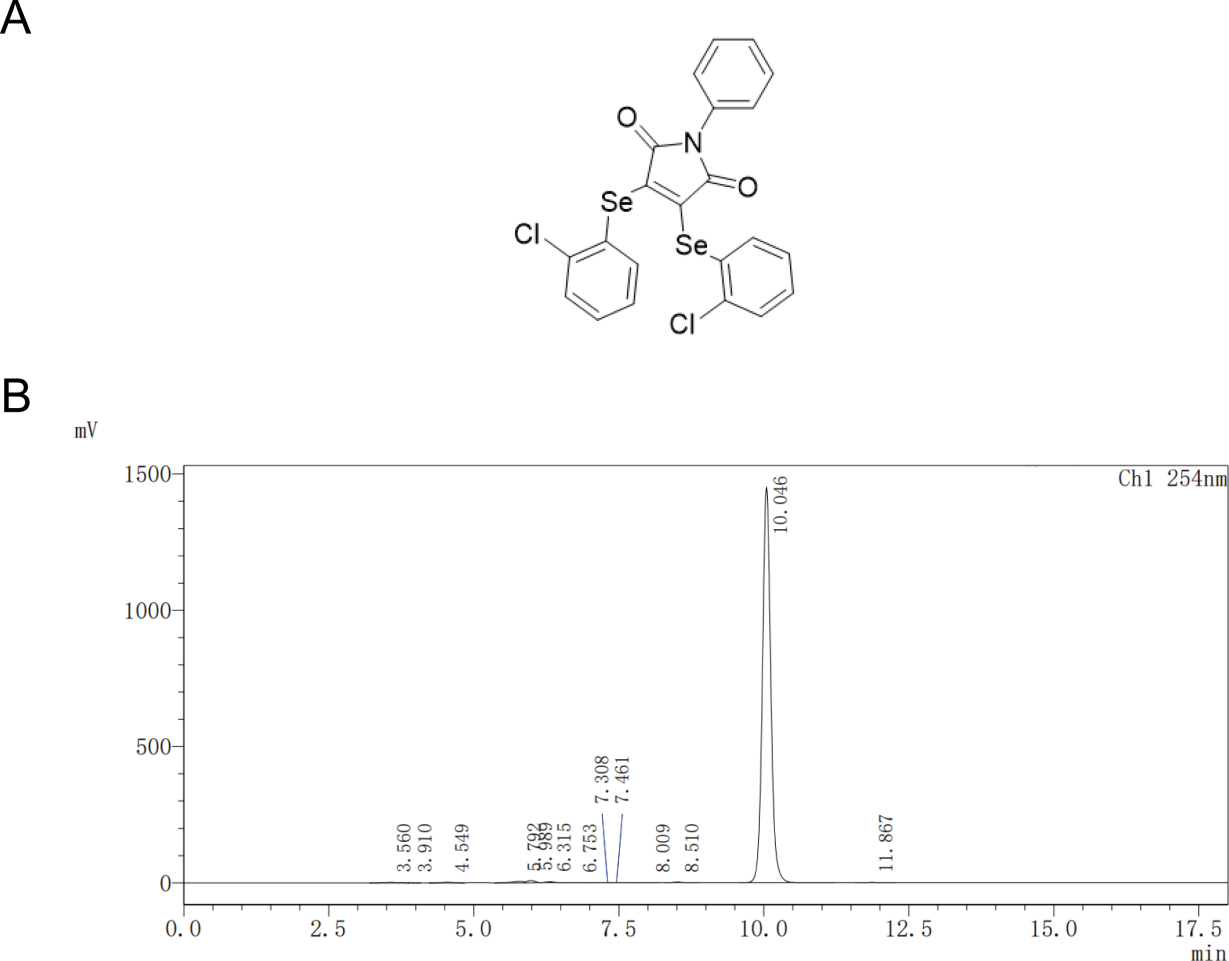
(**A**) Chemical structure of CY-158-11 and (**B**) HRMS identification of CY-158-11.

The synthesis process of CY-158-11 (3,4-bis([2-chlorophenyl]selanyl)-1-phenyl-1H-pyrrole-2,5-dione) was performed as described previously (14), and the chemical structure is shown in Fig. 1A. The compound was obtained as a yellow solid (85.2 mg, 80%) and purified through a chromatography column (elution: 10% EtOAc in petroleum ether), mp 136°C–137°C (Fig. 1B), which was synthesized by the Song laboratory at the School of Pharmacy, Wenzhou Medical University. High-resolution mass spectrometry (HRMS; ESI) m/z: [M + H]^+^ calculated for C_22_H_14_Cl_2_NO_2_Se_2_: 553.8732; found: 553.8724. CY-158-11 was dissolved in DMSO as a stock solution (16 mg/mL).

### *In vitro* evaluation for antibacterial activity of CY-158-11

To evaluate its antibacterial activity *in vitro*, we determined its MIC and MBC against a variety of bacteria, including gram-negative and gram-positive bacteria. CY-158-11 showed outstanding antibacterial activity against gram-positive bacteria, *Staphylococcus* in particular with MICs ranging from 4 to 16 µg/mL (Table 2), compared to its effect on gram-negative bacteria. However, the MICs for gram-negative bacteria, including *K. pneumoniae* (NTUH-K2044), *P. aeruginosa* (PAO1), and *E. coli* (ATCC25922), were more than 128 µg/mL. The MBCs of CY-158-11 against methicillin-sensitive *Staphylococcus aureus* (MSSA) and methicillin-resistant *Staphylococcus aureus* (MRSA), as well as other bacteria, were all more than 128 µg/mL (>32 MIC), indicating that bacteria were tolerant to this compound (15). Bacteriostatic activity has been defined as a ratio of MBC to MIC of >4, while it is considered a bactericidal agent when the ratio is ≤2 (16). Combined with the MBC, we confirm that CY-158-11 is a bacteriostat based on its MBC and MIC values.

**TABLE 2:** The activity of CY-158-11 against pathogenic bacteria

Strains	MIC (μg/mL)	MBC (μg/mL)
*S. aureus*		
MSSA		
ATCC25923	8	>128
ATCC29213	16	>128
Newman	4	>128
NCTC8325	4	>128
JP21	4	>128
SA113	4	>128
MRSA		
Mu50	4	>128
MW2	4	>128
N315	4	>128
USA300	4	>128
JP30	4	>128
MR255	16	>128
*S. epidermidis*		
RP62A	8	>128
ATCC12228	4	>128
SE1457	4	>128
*K. pneumoniae*		
NTUH-K2044	>128	>128
*P. aeruginosa*		
PAO1	>128	>128
*E. coli*		
ATCC25922	>128	>128

**Fig 2:**
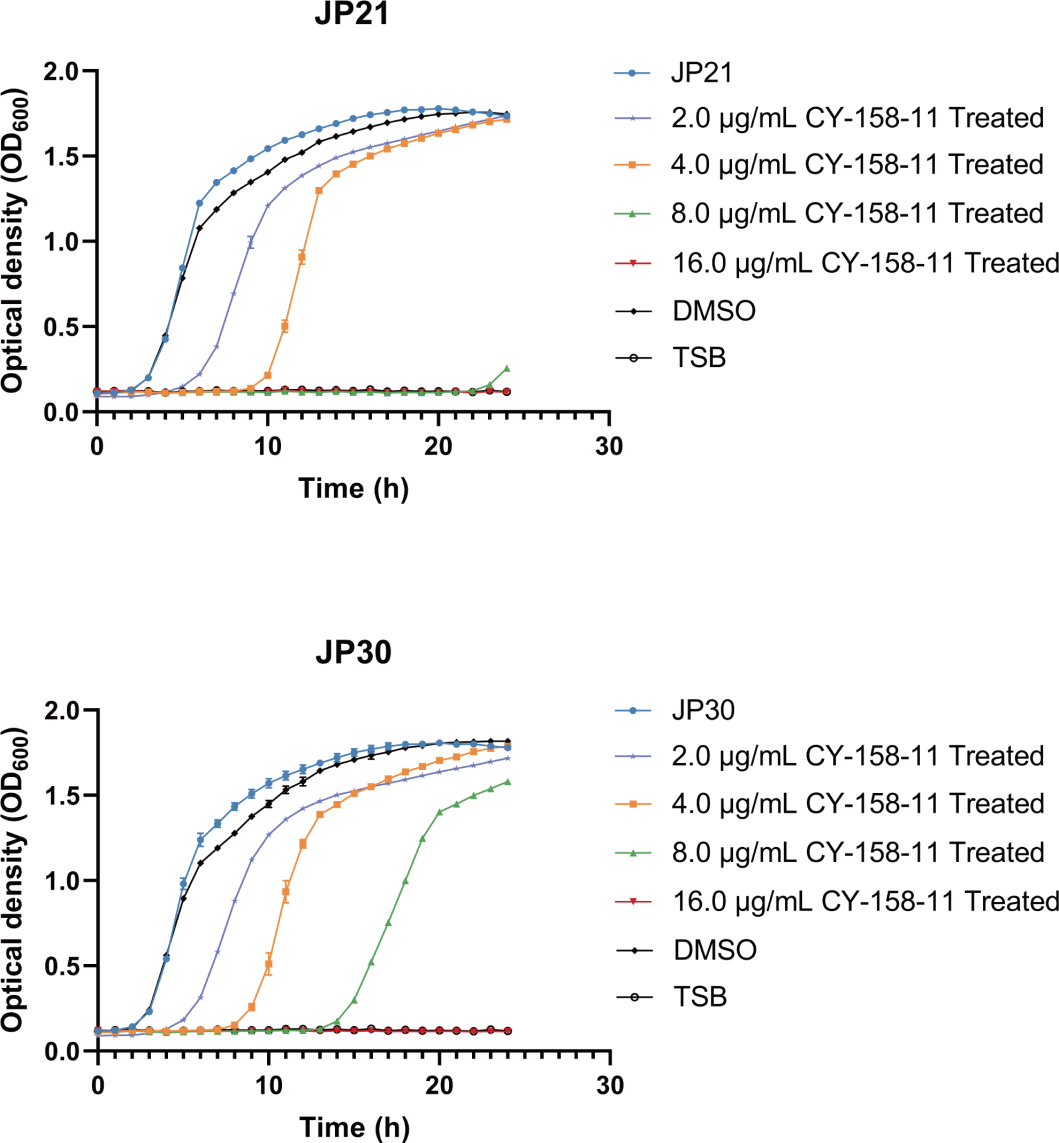
Growth curves of *S. aureus* strains treated with CY-158-11. Strains were cultured with 2.0, 4.0, 8.0, and 16.0 mg/mL or without CY-158-11. Trypticase soy broth was used as a negative control. DMSO was used as a control to exclude the influence of solvent on bacterial growth.

**Fig 3:**
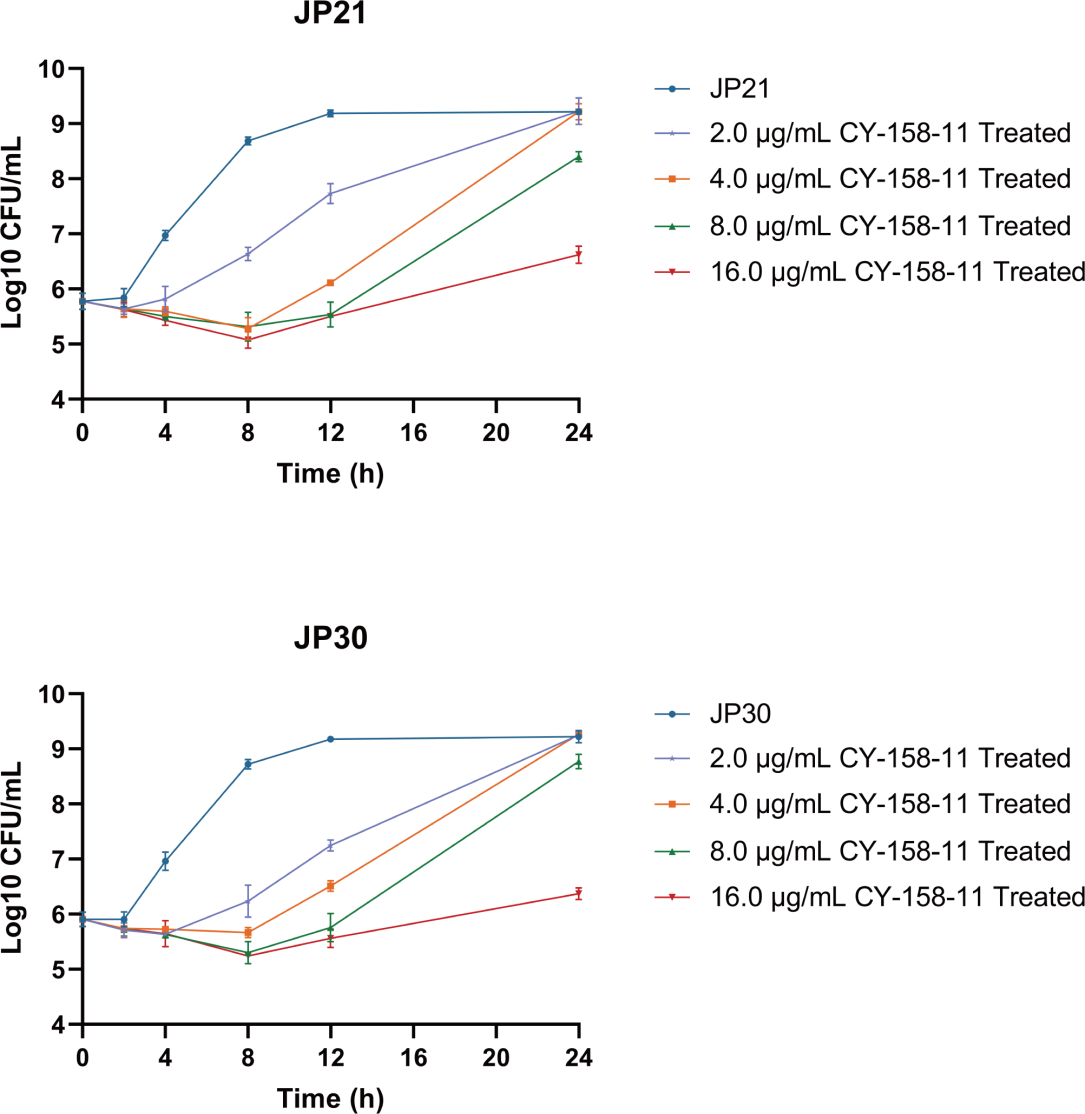
Time-kill curves of *S. aureus* strains treated with CY-158-11. Strains were cultured with 2.0, 4.0, 8.0, and 16.0 mg/mL or without CY-158-11.

We also assessed the growth inhibitory effects of CY-15811 against MSSA JP21 and MRSA JP30. The growth curves illustrated that 2 µg/mL (1/2× MIC) CY-158-11 influenced the growth of *S. aureus*, increasing the time taken for bacteria to reach the stationary phase (Fig. 2). Especially at a concentration of 16 µg/mL (4× MIC), CY-158-11 completely inhibited the growth of the strains. The period of lag phase was prolonged in the presence of 4 µg/mL (1× MIC) followed by a recovery of bacterial growth after 10 h. This differed slightly from the results of the micro-broth dilution method, potentially due to the use of TSB, which provided better nutrition compared to CAMHB, combined with the vigorous shaking that gives the bacteria sufficient exposure to oxygen, both of which allowed these two strains to grow more rapidly. Furthermore, we tested the bacteria viability of JP21 and JP30 exposed to various concentrations of CY-158-11 at different times (Fig. 3). Strong antibacterial effects were observed with CY-158-11 concentrations ≥1× MIC, and 16 µg/mL (4× MIC) CY-158-11 could kill most bacteria within 8 h. What is more, fourfold MIC of CY-158-11 still showed stronger bacteriostatic and even bactericidal activity in the presence of high density of *S. aureus* cells (approximately 10^7^ CFU/mL) (Fig. S1). Since the rate and extent of killing increased with progressively higher antibacterial concentrations, this compound exhibited concentration-dependent bacterial killing.

### CY-158-11 does not induce drug resistance in *S. aureus*

**Fig 4:**
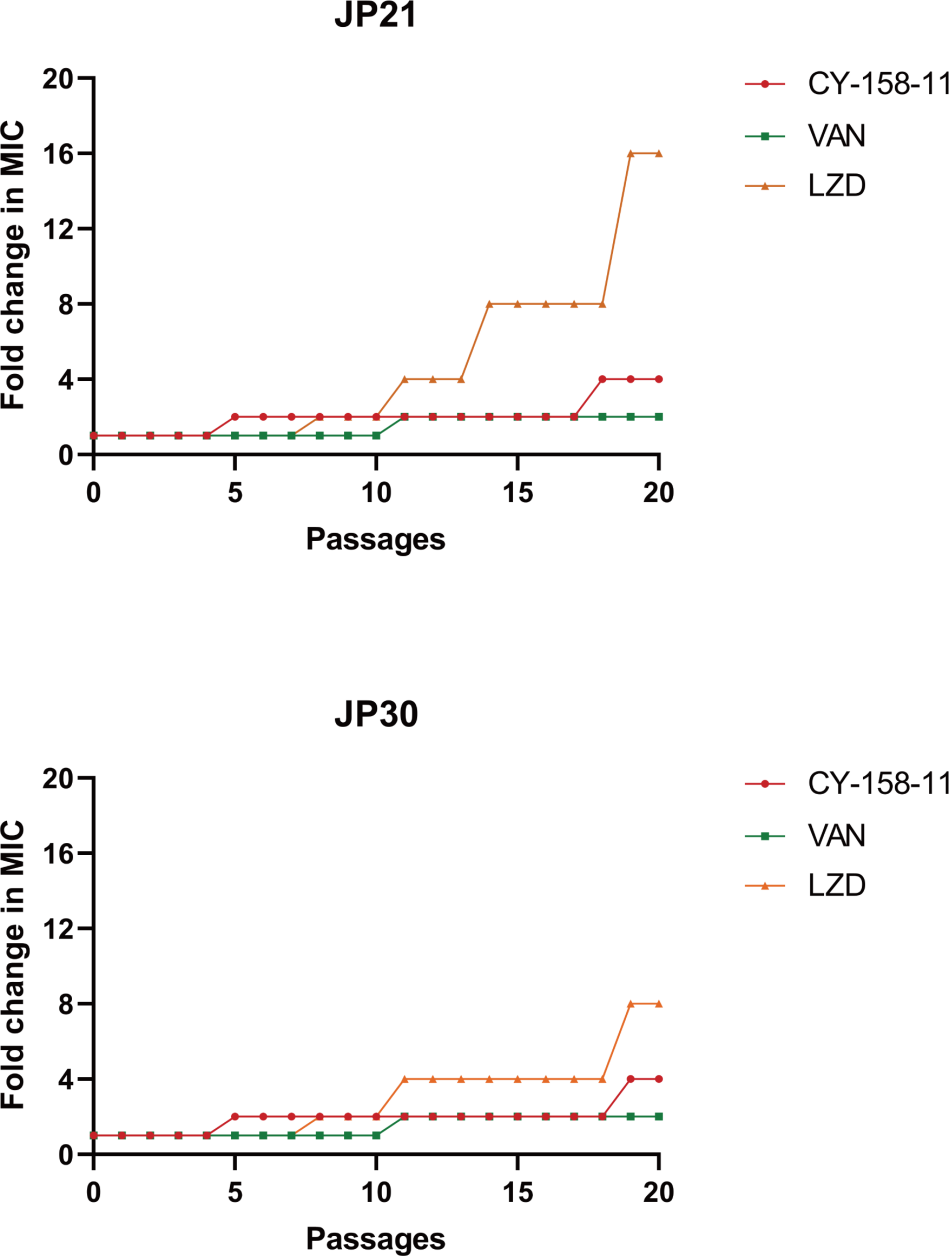
Resistance development of *S. aureus* strains to CY-158-11, vancomycin (VAN), and linezolid (LZD). Values indicate fold changes (in log2) in MIC relative to the MIC of the first passage.

Whether the drug develops resistance is crucial in understanding the long-term efficacy and potential limitations of these drugs in combating *S. aureus* infections. Hence, we investigated the propensity of *S. aureus* to develop resistance to CY-158-11 and other antibiotics by constantly exposing bacteria to a 0.5× MIC concentration of drugs for 20 days. Vancomycin and linezolid are indeed among the mainstays in treating infections caused by resistant gram-positive bacteria. Compared to the 2-fold and 8-/16-fold increase in MIC exhibited by vancomycin and linezolid, respectively, the MIC of CY-158-11 merely increased 4-fold, demonstrating that CY-158-11 displayed relatively low levels of resistance development (Fig. 4). Additionally, the strains that exhibited an elevated MIC showed no change in susceptibility to other drugs, except for the linezolid-induced group which demonstrated decreased susceptibility to vancomycin. These findings imply that unlike most antimicrobials, where resistance to antibiotics is always likely to occur, CY-158-11 holds promise as a leading antibacterial agent against *S. aureus*.

### CY-158-11 disrupts the membrane integrity of *S. aureus*

**Fig 5:**
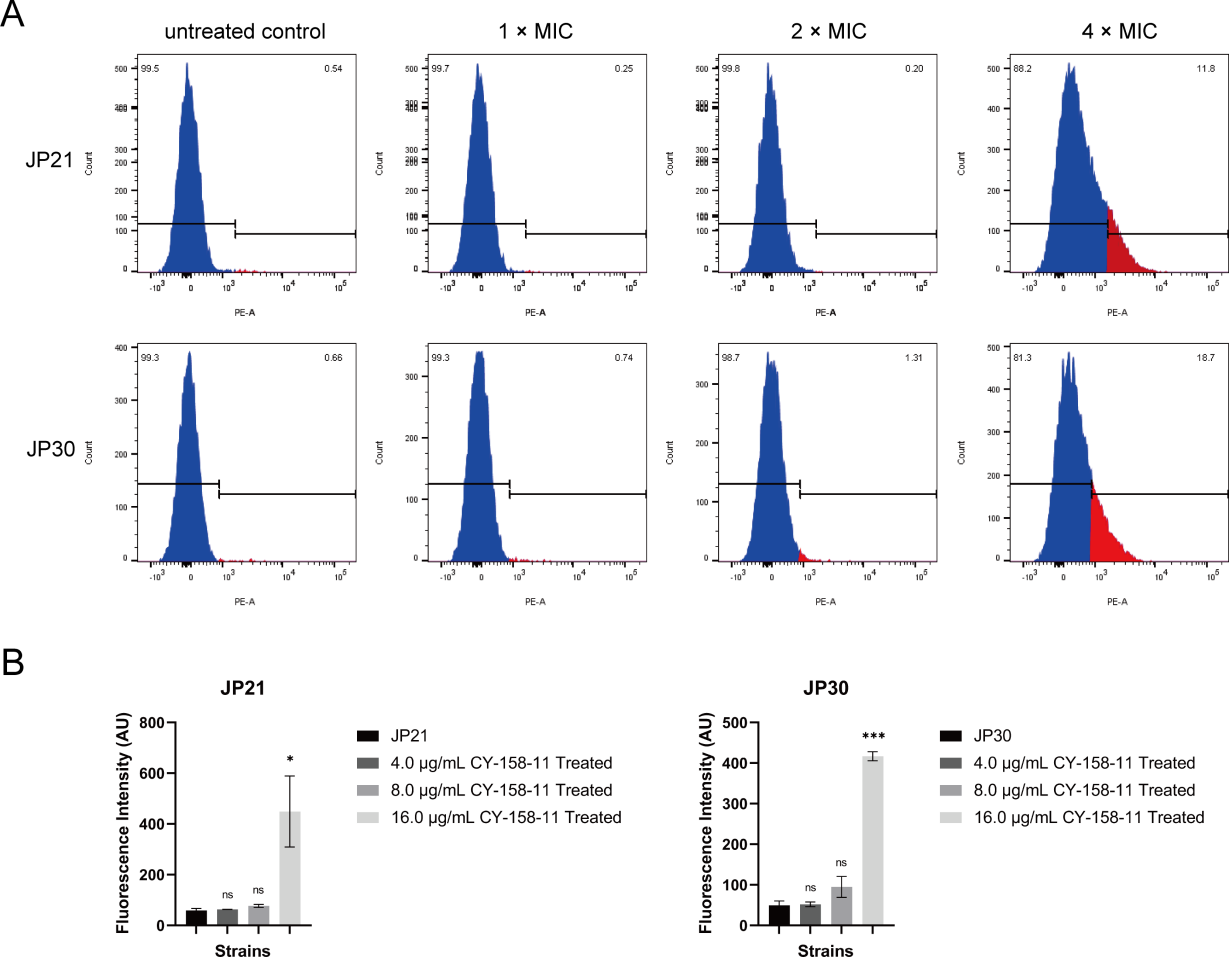
CY-158-11 disrupts the integrity of the bacterial cell membrane. (**A**) Flow cytometric analysis of PI staining in *S. aureus* cells treated with various concentrations of CY-158-11. (**B**) Changes in membrane integrity were indicated by PI fluorescence in *S. aureus* cells. The data were expressed as the percentages of membrane-compromised bacteria in at least 10,000 cells and presented as mean ± SD (*n* = 3 per group). ns, non-significant; ^*^P < 0.05; ^***^P < 0.001.

To evaluate whether the enhanced antibacterial effect of CY-158-11 was associated with alteration of bacterial membrane integrity, we quantified the uptake of propidium iodide in JP21 and JP30 following the treatment of CY-158-11 to the bacteria for 4 h. PI was a membrane-impermeable fluorescent dye that was inserted into double-stranded DNA and was generally used to evaluate the bacterial viability (17). For the control without CY-158-11, the percentage of PI-positive *S. aureus* cells was very low, indicating that the bacterial cell membranes were intact. The proportion of *S. aureus* cells that are PI positive rose along with the rise in CY-158-11. The treatment of CY-158-11 at 16 µg/mL (4× MIC) on *S. aureus* significantly increased the uptake of PI dye, as assessed by an increase in PI fluorescence, suggesting a membrane-lytic ability of the compound (Fig. 5A and B).

### CY-158-11 dissipates the membrane potential of *S. aureus*

**Fig 6:**
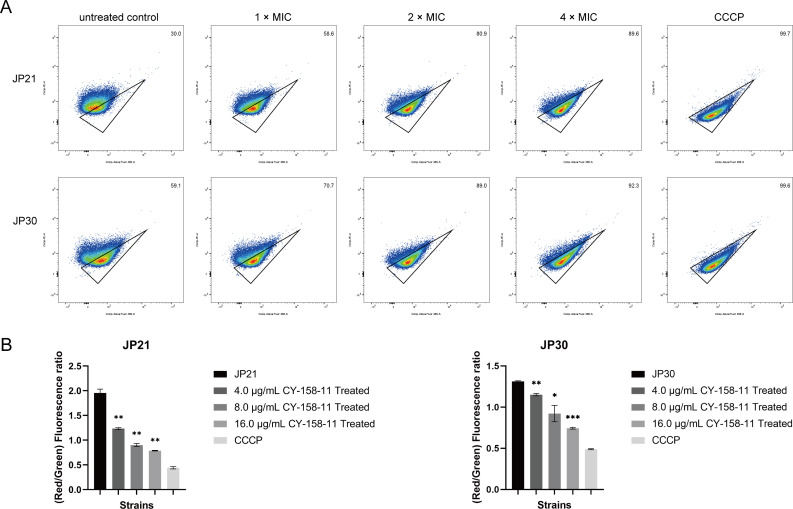
CY-158-11 depolarizes the bacterial cell membrane. (**A**) Flow cytometric analysis of DiOC_2_(3) staining in *S. aureus* cells exposed to CCCP or various concentrations of CY-158-11. (**B**) Changes in membrane potential were calculated as the ratio of cells that exhibited red fluorescence to cells that displayed green fluorescence and were expressed as a percentage relative to the untreated control. The data shown were the mean ± SD (*n* = 3 per group). ^*^P < 0.05; ^**^P < 0.01; ^***^P < 0.001.

To investigate the ability of CY-158-11 to depolarize bacterial membranes, we observed the degree to which the fluorescence shifted its fluorescent properties from red fluorescence to green released by DiOC_2_(3). When the bacterial cytoplasmic membrane was disrupted, the membrane potential dissipates *S. aureus*, and the dye molecules aggregated, resulting in a fluorescence increase (18). CY-158-11 at 16 µg/mL (4× MIC) showed a remarkable increase in green fluorescence intensity, which was very similar to the CCCP-treated controls (JP21: 89.6% vs 99.7%; JP30: 92.3% vs 99.6%) (Fig. 6A). When cells were treated with CY-158-11, the red and green fluorescence ratio, represented as membrane potential, decreased dose dependently. In two *S*. *aureus* strains, damaged cells treated with CY-158-11 at 16 µg/mL (4× MIC) exhibited 59.7% and 43.3% decreases in membrane potential induced by cell membrane depolarization, respectively (Fig. 6B). These results verified a significant membrane depolarization effect of CY-158-11 in *S. aureus*.

### CY-158-11 destructs the cell wall and membrane of *S. aureus*

**Fig 7:**
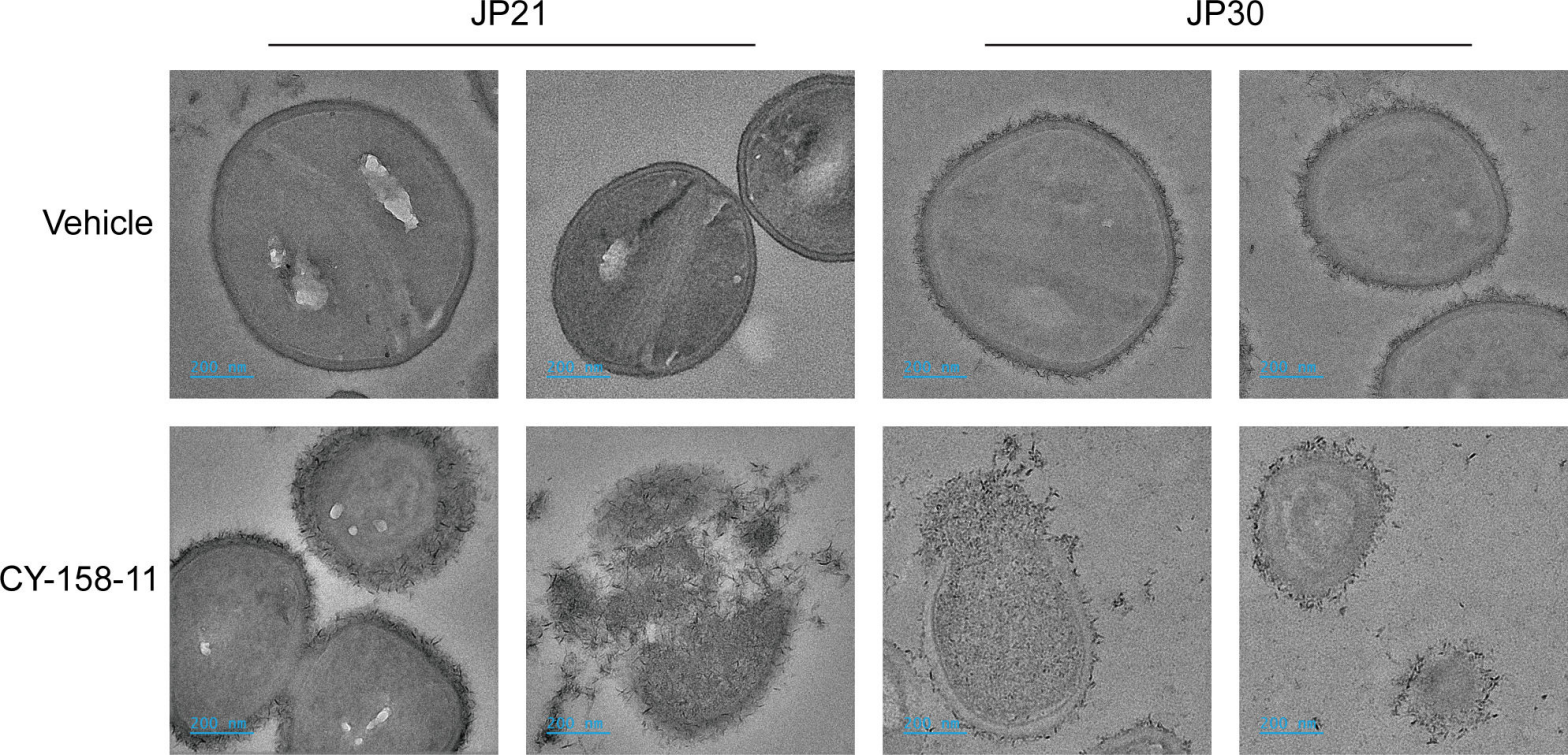
Transmission electron microscopy analysis of *S. aureus* cells treated with CY-158-11. The micrographs were acquired at a final magnification of %10,000 and are representative of the bacterial ultrastructure observed in >10 cells. The scale bars shown represent 200 nm.

A parallel experiment to visualize the morphological structure of the cell surfaces by transmission electron microscopy further revealed that the bacteria exposed to CY-158-11 at 16 µg/mL (4× MIC) were associated with significant rupture of the cell wall and membrane, as evidenced by the leakage of intracellular content (Fig. 7). Relatively speaking, the untreated *S. aureus* JP21 and JP30 cells showed a normal and intact structure. These findings suggested that CY-158-11 possesses a membrane-lytic capability, though the detailed structural interactions between CY-158-11 and bacterial membrane remain to be elucidated.

### CY-158-11 does not impair the cells and mice at an effective concentration

**Fig 8:**
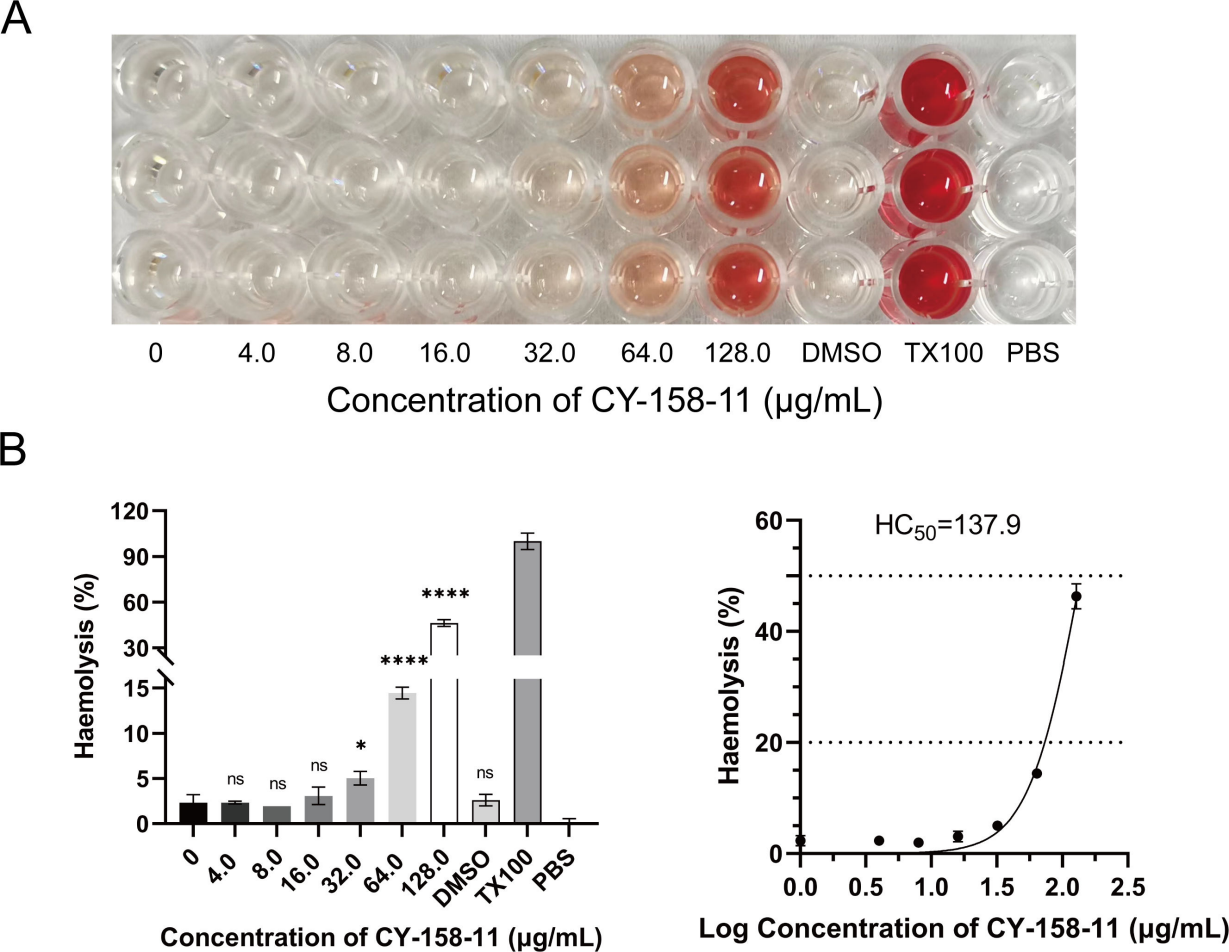
Hemolytic activity of various concentrations of CY-158-11 on human red blood cells. (**A**) Representative images for hRBCs hemolysis in response to CY-158-11. (**B**) The percentages of hemolysis and 50% hemolytic concentrations of CY-158–11. ns, non-significant; *P < 0.05; ****P < 0.0001.

**Fig 9:**
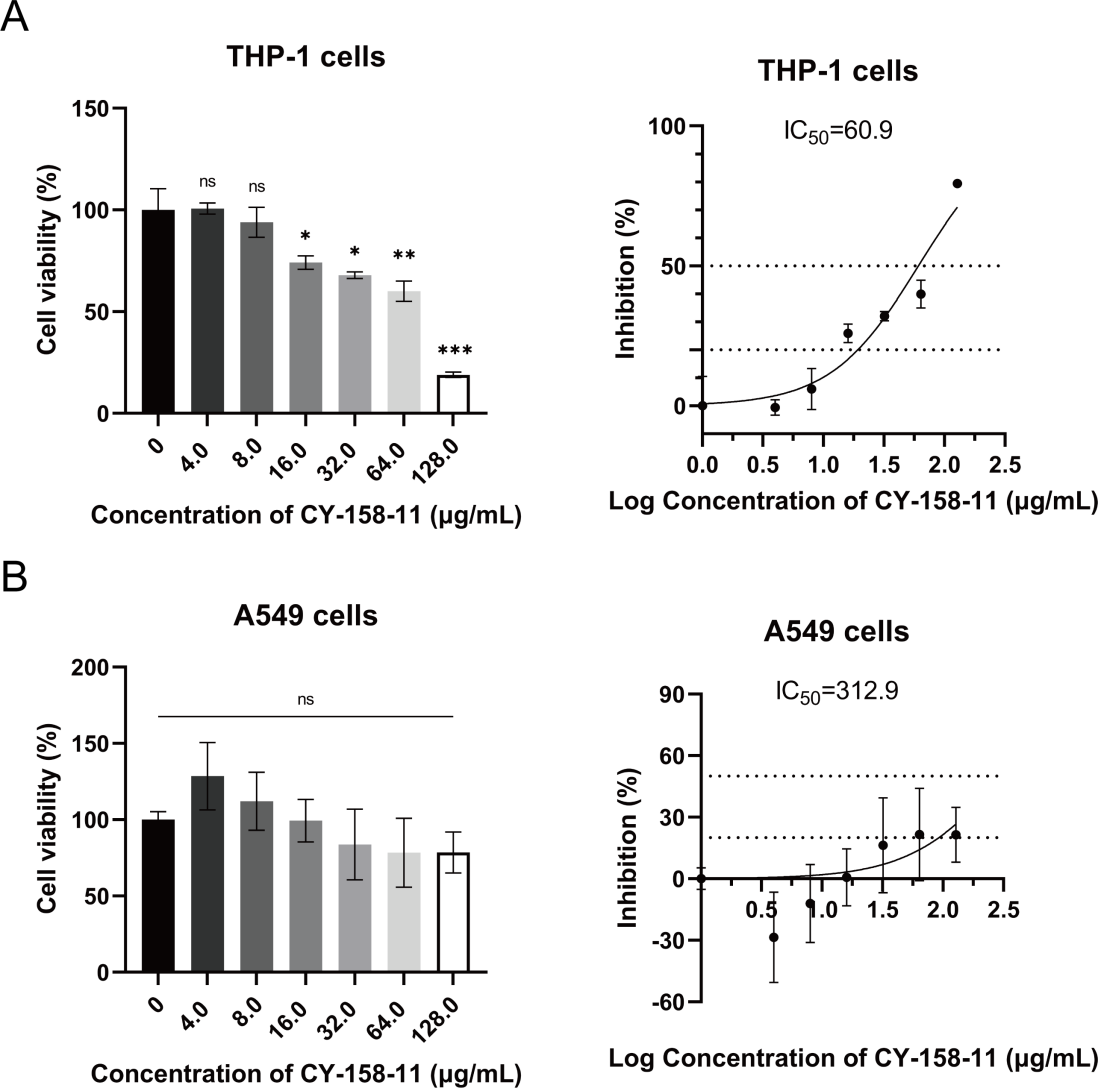
Cytotoxicity of various concentrations of CY-158-11 on (**A**) THP-1 and (**B**) A549 cells. The data are shown as the percentages of cell viability and 50% inhibitory concentrations of CY-158-11 against cells.

Numerous natural or synthetic compounds eliminate bacteria by specifically attacking the cell membrane (19). However, it is important to note that they can also unintentionally interfere with the cytoplasmic membrane present in eukaryotic cells. To determine whether there was any interaction between CY-158-11 and the RBC cell membrane, we measured hemoglobin release from hRBCs and found that CY-158-11 showed no significant hemolytic activity on RBC membranes below a concentration of 32 µg/mL, which was eightfold greater than its MIC for *S. aureus* (Fig. 8A). The hemolytic concentration of 50% (HC50) was measured to be 137.9 µg/mL (Fig. 8B). In addition, similar results were identified in CY-158-11-treated THP-1 and A549 cells. CY-158-11 inhibited the viability of the THP-1 (Fig. 9A) and A549 (Fig. 9B) cell lines in a dose-dependent manner, with 50% inhibitory concentrations of 60.9 and 312.9 µg/mL, respectively. The selectivity index values against hRBCs, THP-1, and A549 cell lines for *S. aureus* ranged from 8.62 to 34.48, 3.81 to 15.23, and 19.56 to 78.23, respectively. A549 cells generally displayed higher SI values compared to hRBCs and THP-1 cells. SI >1 regarded to be more toxic to bacterial cells than mammalian cells (20). Therefore, CY-158-11 exhibited good selectivity index (>3.0) for these three types of cells, providing a large safety margin between the concentration of CY-158-11 that was able to kill the *S. aureus* and the concentration that was toxic to mammalian cells in this case (21).

**Fig 10:**
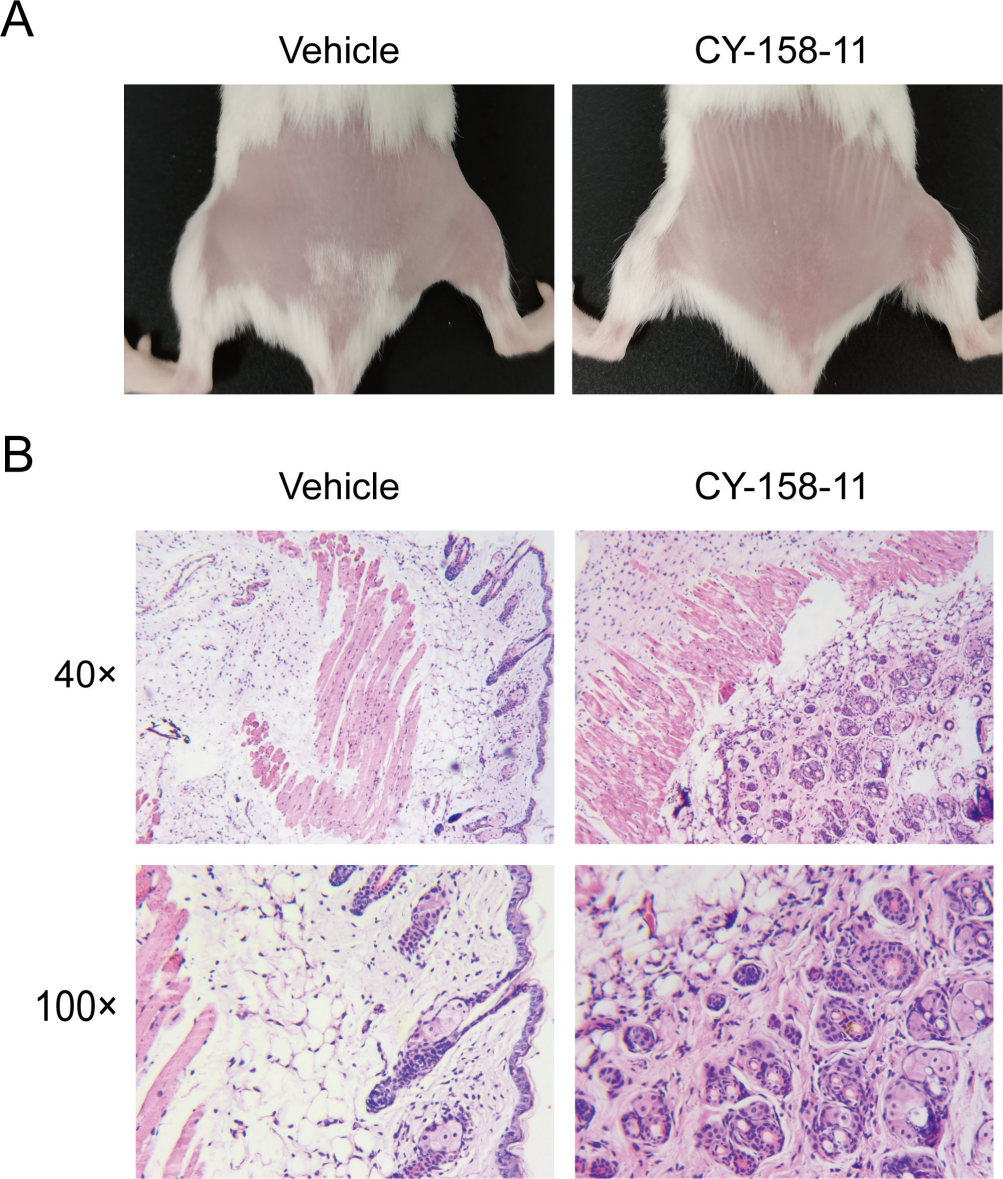
The safety of CY-158-11 to the skin of mice. (**A**) Skin conditions of mice after CY-158-11 treatment on day 4. (**B**) Pathological sections of representative mice between the normal saline treatment group and the CY-158-11 treatment group.

**Fig 11:**
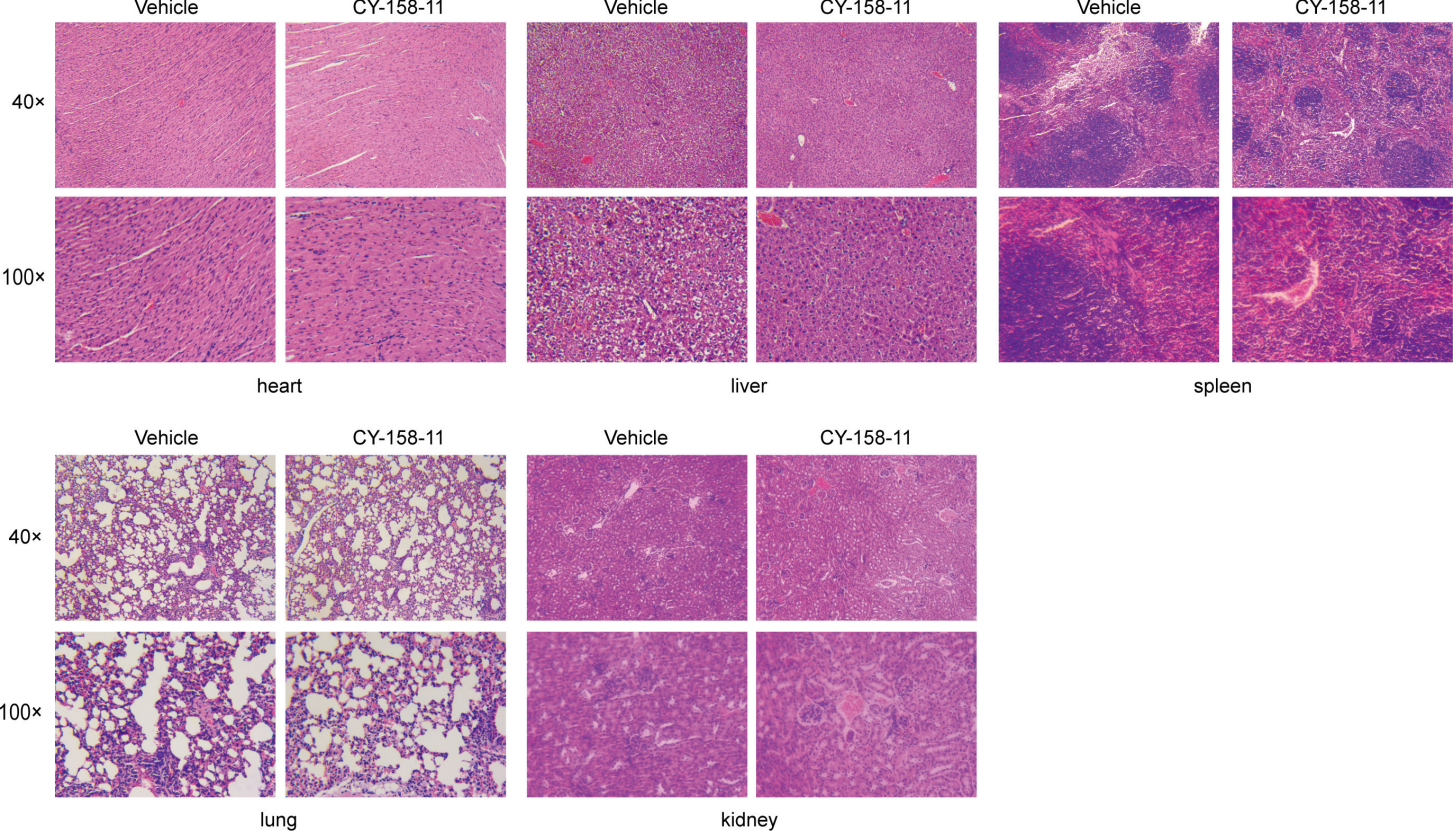
The biosafety evaluation of CY-158-11 *in vivo*. Pathological sections of internal organs in representative mice between the normal saline treatment group and the CY-158-11 treatment group.

Moreover, we conducted experiments to identify the potential toxicity of CY-158-11 in mice. After three injections of 100 µL of 16 µg/mL CY-158-11, we observed that the epidermis of the mice remained intact, the dermis did not show any noticeable damage, and there was no obvious inflammatory cell infiltration (Fig. 10A and B). Similarly, mice that were administered 100 µL of 16 µg/mL CY-158-11 through their tail vein did not exhibit any noticeable pathological alterations in their internal organs, including heart, liver, spleen, lung, and kidney (Fig. 11). Altogether, it appears that CY-158-11 did not induce any damage to the skin and viscera of mice at the effective concentration of 16 µg/mL (4× MIC).

### *In vivo* validation using mouse skin abscess model

**Fig 12:**
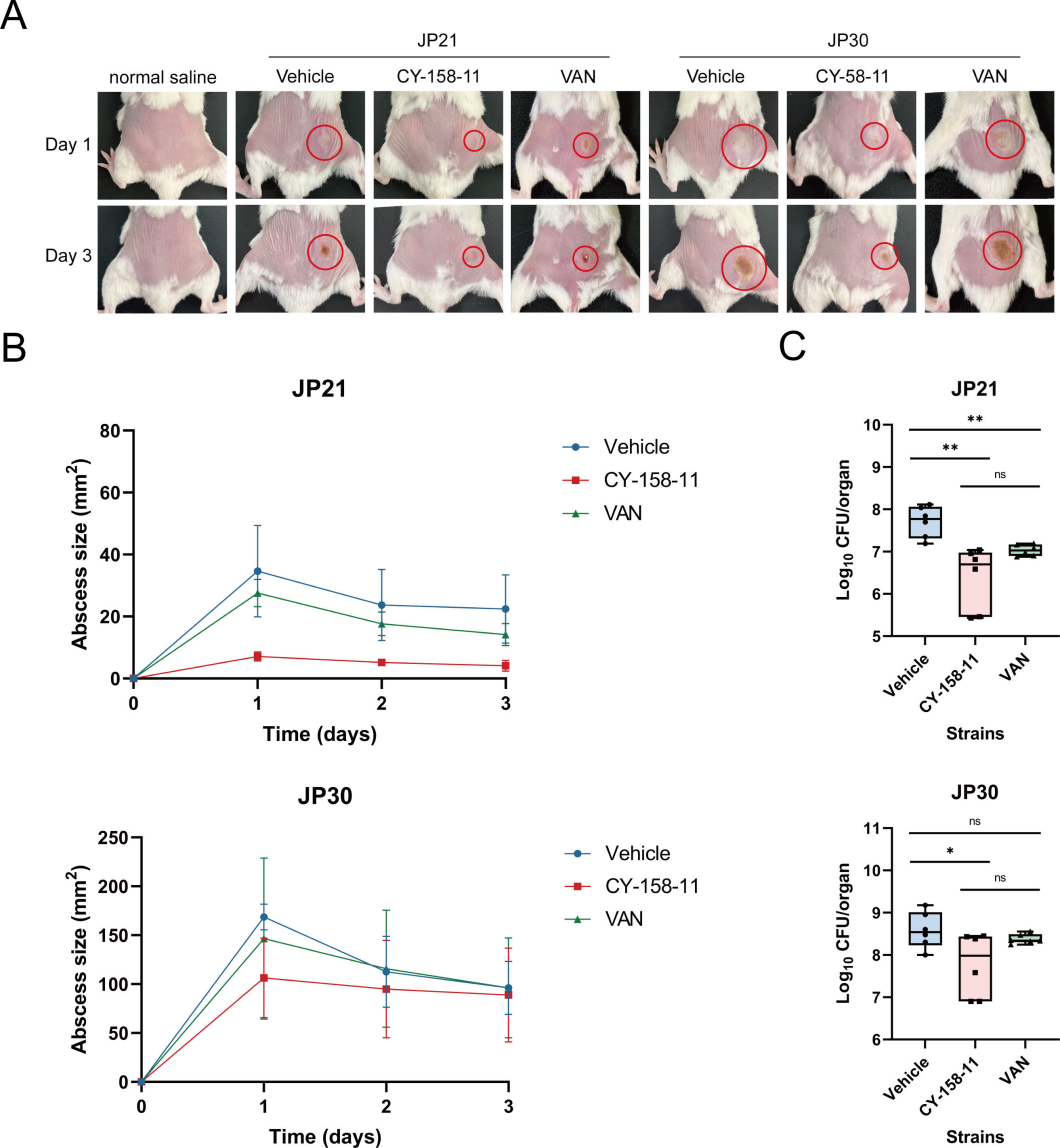
CY-158-11 therapy in the mouse abscess model constructed by *S. aureus*. (**A**) The image of representative abscesses before and after CY-158-11 treated on day 1 and day 3 after infection. (**B**) Daily changes of skin abscess area in mice with CY-158-11 treatment or not. (**C**) Comparison of bacterial colonies in mice skin abscesses of the normal saline treatment group and the CY-158-11 treatment group. ns, non-significant; ^*^P < 0.05; ^**^P < 0.01. VAN, vancomycin.

*S. aureus* is the predominant pathogen responsible for purulent skin and soft tissue infections (22). By verifying the capacity of CY-158-11 in promoting the bacteriostatic activity against *S. aureus in vitro*, we immediately sought to validate its efficacy *in vivo* using a mouse model of skin infection induced by subcutaneously injecting a high density (approximately 10^8^ CFU/mouse) of JP21 and JP30. Abscesses of ≈34.6 mm^2^ (JP21) and 168.7 mm^2^ (JP30) were observed in the normal saline treatment groups on day 1. Notably, the size of the abscesses was significantly reduced after treatment with 0.08 mg/kg CY-158-11 by 79.5% and 36.7%, respectively (Fig. 12A and B). Besides, the results of bacteria counts displayed comparable trends. The single-dose treatment of CY-158-11 reduced *S. aureus* JP21 and JP30 numbers by 95.3% and 84.5%, respectively. Therefore, the number of *S. aureus* in the wound decreased by more than 10 times, compared with the normal saline treatment group (Fig. 12C). Furthermore, 0.01 mg/kg vancomycin has a poor therapeutic effect; comparatively speaking, *S. aureus*-infected abscess tissues treated with vancomycin were larger than abscess tissues treated with CY-158-11, although there was no significant difference in bacterial burden. In accordance with the description above, images showed that CY-158-11 remarkably reduced abscess size and improved the bacteria removal rate from the infection site. These results suggested that CY-158-11 showed a good therapeutic effect and could be used to treat skin infections caused by *S. aureus* in mice.

## DISCUSSION

*S. aureus* is a widespread pathogen accountable for infections in both hospitals and communities. Its resistance to multiple antibiotics poses a great challenge in the realm of treatment (18). However, the discovery and advancement of innovative categories of antibacterial drugs, on the other hand, are experiencing a deceleration (23). Previous studies have confirmed that a novel diselenide-maleimide hybrid, CY-158-11, can mitigate the biofilm formation of *S. aureus* (13). In this study, CY-158-11 exhibited the capacity against all *S. aureus* strains tested *in vitro* and *in vivo*; however, it showed no obvious activity against gram-negative bacteria. The selective activity of the drug against gram-positive and gram-negative bacteria remains to be explored, which may be related to structural differences (24, 25). Along with carbon, hydrogen, nitrogen, and oxygen, chlorine is one of the most common atoms in small-molecule compounds. Replacing a hydrogen atom (R = H) with a chlorine atom (R = Cl) may seem simple, but it significantly increases potency (26). We have found that the compound YH7 also exhibited remarkable antimicrobial activity against *S. aureus* in planktonic cultures and biofilms (27). The compound YH7 with a similar structure, but lacking two chlorine atoms compared to CY-158-11, may cause its MIC (16 µg/mL) to be higher. Consequently, CY-158-11 has a fourfold higher antimicrobial activity than YH7. The possible explanation for this is that the two chlorine atoms not only increased the lipophilicity but also their electron-withdrawing properties (28). Further work will be necessary to confirm these explanations.

Here, we attempted to report the antibacterial effect and its mechanism of CY-158-11 against *S. aureus* isolates. The time-kill kinetics data indicated that the effect of CY-158-11 was probably bacteriostatic rather than bactericidal, which was consistent with the MBC/MIC value. Additionally, antibiotic misuse produces a broad spectrum of selective pressure and increases the potential for drug resistance (29), leading to the spread of resistance mechanisms between bacteria and posing a challenging issue in therapeutic therapy. Vancomycin is the first-line treatment drug as per the treatment guidelines for MRSA infections (30). However, with the widespread use of vancomycin, vancomycin-resistant MRSA appears, which brings tremendous difficulties to clinical treatment (31, 32). Linezolid is a crucial therapeutic alternative for the treatment of infections caused by glycopeptide- and beta-lactam-resistant gram-positive organisms (33). It has been specifically developed for the treatment of infections caused by MRSA, especially for lung infections and certain parenchymal organ infections, whose efficacy is significantly superior to that of vancomycin (34). Unfortunately, long-term treatment with linezolid tends to develop resistance, which is consistent with the results of our resistance-inducing assay. In addition, the decreased susceptibility of *S. aureus* to linezolid appears to be accompanied by a reduced activity toward vancomycin, necessitating further investigation. CY-158-11 displayed low levels of resistance development, intermediate between vancomycin and linezolid, and lacked cross-resistance with other antimicrobials, highlighting its potential to combat *S. aureus*.

As seen by the holes and breakage on the surface of the bacteria treated with CY-158-11, the bacterial first line of defense was defeated, which was confirmed by the TEM images. After that, PI entered the cell membrane, implying that CY-158-11 can kill bacteria by interacting with and permeabilizing bacterial cytoplasmic membranes, contributing to bacterial cells rupturing, and then intracellular nucleic acids, proteins, and other substances leaking (35). Membrane potential is determined by the difference between the interior and exterior electric potential in viable cells (36), regulating a wide range of bacterial physiology and behaviors, such as pH homeostasis, membrane transport, motility, antibiotic resistance, cell division, electrical communication, and environmental sensing (37). The decreased membrane potential of *S. aureus* treated with CY-158-11 extracts was indicative of a change in interior and exterior ion concentrations induced by membrane damage. The loss of red fluorescence intensity and the emission of green fluorescence intensity accounted for cell membrane depolarization and bacterial death (38).

CY-158-11 can selectively destroy the cytoplasmic membrane of bacteria without harming that of mammalian cells. In our hemolysis and cytotoxicity assay, the results revealed that CY-158-11 in a certain range of concentrations was non-toxic to human red blood cells, THP-1 cells, and A549 cells. The cell line and solvent are not responsible for the toxicity of high CY-158-11 concentrations, and thus, we speculate that the toxicity is dose dependent. CY-158-11 concentrations for half-maximal inhibition values were well higher than the effective antibacterial concentration. To identify the *in vivo* effects of CY-158-11, we used a mouse skin abscess model. According to the results, a single dose of 0.08 mg/kg could produce a therapeutic effect on skin wound infection in mice. Besides, we found through preliminary experiments that this dose of CY-158-11 is also safe for mice, which was also confirmed in the skin irritation test and systemic toxicity test. In general, CY-158-11, which exerted an excellent antibacterial effect against *S. aureus*, was below the upper limit of the safe concentration, demonstrating a great possibility of the clinical application of *S. aureus* infection treatment.

## SUPPLEMENTAL MATERIAL

Figure S1 msphere.00643-24-s0001.docx CY-158-11 inhibits *S. aureus* growth even at a high density.

ASM does not own the copyrights to Supplemental Material that may be linked to, or accessed through, an article. The authors have granted ASM a non-exclusive, world-wide license to publish the Supplemental Material files. Please contact the corresponding author directly for reuse.
