# Supplementary material for: Insights into small-molecule compound CY-158-11 antibacterial activity against Staphylococcus aureus
Source: mSphere. 2024 Sep 23;9(10):e00643-24. doi: 10.1128/msphere.00643-24 (PMC11520288; doi:10.1128/msphere.00643-24)
Supplement: Figure S1 — CY-158-11 inhibits S. aureus growth even at a high density. [file msphere.00643-24-s0001.docx]

**Supplementary Figures**


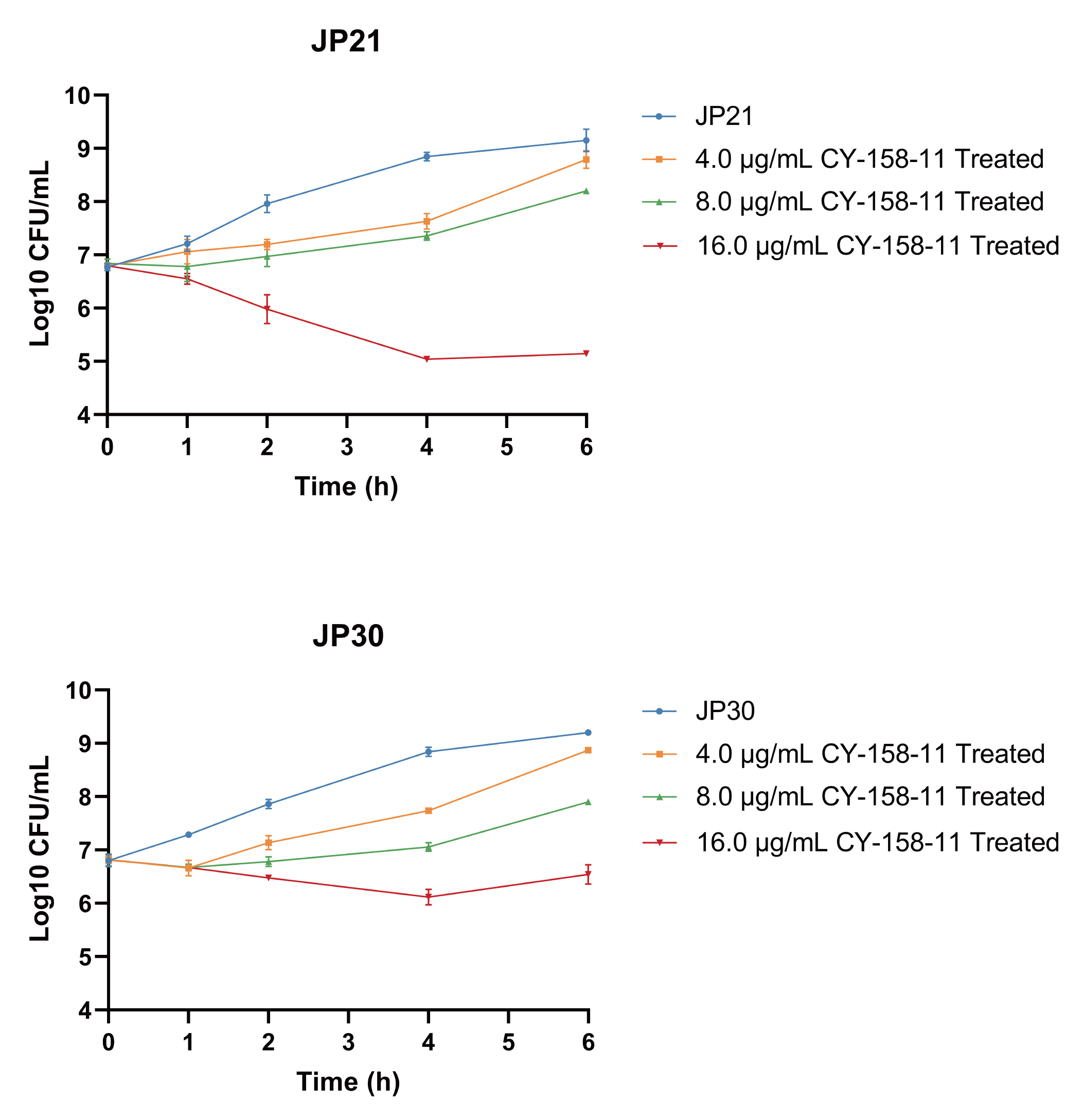


Supplementary Figure 1 | CY-158-11 inhibits *S. aureus* growth even at a high density. Viable cell counts at 6 h timepoint.
